# TARGET Protein: the effect of augmented administration of enteral protein to critically ill adults on clinical outcomes—statistical analysis plan for a cluster randomized, cross-sectional, double cross-over, clinical trial

**DOI:** 10.1186/s13063-025-08759-0

**Published:** 2025-02-06

**Authors:** Sophie Zaloumis, Matthew J. Summers, Jeffrey J. Presneill, Rinaldo Bellomo, Lee-anne S. Chapple, Marianne J. Chapman, Adam M. Deane, Suzie Ferrie, Craig French, Sally Hurford, Nima Kakho, Matthew J. Maiden, Stephanie N. O’Connor, Sandra L. Peake, Emma J. Ridley, An Tran-Duy, Patricia J. Williams, Paul J. Young, Amalia Karahalios, on behalf of the TARGET Protein Investigators, the Australian, New Zealand Intensive Care Society Clinical Trials Group

**Affiliations:** 1 Centre for Epidemiology and Biostatistics, Melbourne School of Population and Global Health, Faculty of Medicine, Dentistry and Health Sciences, University of Melbourne, Melbourne, VIC Australia; 2 Methods and Implementation Support for Clinical and Health (MISCH) Research Hub, Faculty of Medicine, Dentistry, and Health Sciences, University of Melbourne, Melbourne, VIC Australia; 3 Intensive Care Unit, Royal Adelaide Hospital, Adelaide, South Australia Australia; 4 Discipline of Acute Care Medicine, The University of Adelaide, Adelaide, South Australia Australia; 5 Australian and New Zealand Intensive Care Research Centre, Monash University, Melbourne, VIC Australia; 6 Department of Critical Care, The University of Melbourne, Melbourne, Australia; 7 Intensive Care Unit, Royal Melbourne Hospital, Parkville, VIC Australia; 8 Intensive Care Unit, Austin Health, Heidelberg, VIC Australia; 9 Centre for Research Excellence in Translating Nutritional Science to Good Health, National Health and Medical Research Council of Australia, University of Adelaide, Adelaide, South Australia Australia; 10 Department of Nutrition & Dietetics, Royal Prince Alfred Hospital, Camperdown, NSW Australia; 11 Faculty of Medicine and Health, University of Sydney, Sydney, Australia; 12 Intensive Care Unit, Sunshine Hospital, Melbourne, VIC Australia; 13 Medical Research Institute of New Zealand, Wellington, New Zealand; 14 Intensive Care Unit, University Hospital Geelong, Geelong, VIC Australia; 15 Intensive Care Unit, The Queen Elizabeth Hospital, Woodville South, South Australia Australia; 16 Dietetics and Nutrition, Alfred Hospital, Melbourne, VIC Australia; 17 School of Population and Global Health, Centre for Health Policy, The University of Melbourne, MelbourneMelbourne, VIC Australia; 18 Intensive Care Unit, Wellington Hospital, Wellington, New Zealand

**Keywords:** Intensive care, Dietary protein, Cluster-randomized, Cross-over, Trial

## Abstract

**Background:**

The TARGET Protein trial will evaluate the effect of greater enteral protein delivery (augmented protein) on clinical outcomes of critically ill adult patients when compared to usual care.

**Objective:**

To describe the statistical analysis plan for the TARGET Protein trial.

**Methods:**

TARGET Protein is a cluster randomized, cross-sectional, double cross-over, open-label, registry-embedded, pragmatic clinical trial conducted across Australia and New Zealand. The trial randomized eight intensive care units (ICU) to receive enteral formula containing either higher dose enteral protein (augmented protein) or usual dose protein in a 1:1 ratio. Each ICU received one trial formula for a 3-month period and then switched to the alternate formulae. This sequence was repeated, for a total trial length of 12 months. The primary outcome is the number of days free of the index hospital and alive at day 90. Secondary outcomes include proportion of patients alive at day 90, survivor-only analysis of days free of the index hospital at day 90, duration of invasive ventilation, ICU and hospital length of stay, incidence of tracheostomy insertion, renal replacement therapy, and discharge destination. The statistical methods and models which will be used to estimate the effects for the primary and secondary outcomes are described. All statistical models will account for the cluster-randomized cross-over design to ensure correct estimation of the 95% confidence intervals. Trial enrolment is complete with 3412 patients enrolled. Data linkage is ongoing.

**Conclusion:**

This statistical analysis plan enables transparent reporting of the TARGET Protein trial. It will reduce the risk of potential selective reporting biases.

**Trial registration:**

Australian New Zealand Clinical Trials Registry (ACTRN12621001484831). Registered on November 1, 2021.

## Background

Approximately one in four critically ill patients who receive enteral nutrition (EN) in the intensive care unit (ICU) does not survive to hospital discharge [1]. For those that do survive, rapid and substantial muscle wasting occurs [2, 3], which is strongly associated with adverse patient-centered outcomes [4–6]. International critical care nutrition guidelines recommend protein delivery of at least 1.2 g/kg body weight/day [7, 8]. Critically ill patients typically receive dietary protein at rates < 1.0 g/kg body weight/day [9, 10] and it has been proposed that augmenting dietary protein in critical illness could improve outcomes [11, 12]. However, it is unclear from existing data whether augmenting dietary protein in the ICU improves patient outcomes, with existing meta-analysis indicating that increased protein could reduce mortality by up to 12% or increasing mortality by up to 11% [13, 14].

The TARGET Protein trial is a cluster randomized, cross-sectional, double cross-over, registry-embedded, pragmatic open-label clinical trial with the aim of evaluating the effect of higher enteral protein delivery (augmented protein) on clinical outcomes of critically ill adult patients when compared to usual care [15]. This manuscript describes the statistical analysis plan for the TARGET Protein trial and supersedes the plan provided in the previously published protocol [15] and trial registration (Australian New Zealand Clinical Trials Registry, ACTRN12621001484831).

At least three relevant clinical trials have emerged since the commencement of the TARGET Protein trial. During the recruitment for the TARGET Protein trial, the EFFORT Protein trial was published (25 January 2023) [16]. That trial evaluated the effect of increased protein (≥ 2.2 g/kg/day) compared to usual care (protein ≤ 1.2 g/kg/day) and was ceased with less than one third of the planned cohort recruited at 1301 patients due to slow recruitment consequent upon the COVID-19 pandemic. The revised primary outcome of the EFFORT Protein trial was “time to discharge alive from hospital up to 60 days after ICU admission” and there was no evidence of a between group difference [16]. The authors reported potentially worse outcomes in the sub-group of patients with acute kidney injury [17] and high SOFA score (≥ 9) on admission; however, the authors acknowledged multiplicity of testing with accompanying inflation of type 1 errors. Further, the sub-group with acute kidney injury was defined according to the Kidney Disease: Improving Global Outcomes (KDIGO) creatinine criteria, which include post-randomization biochemical changes up to day 7.

The PRotEin Provision in Critical IllneSs (PRECISe) trial was an investigator-initiated, multi-center, parallel group, blinded, randomized controlled trial evaluating functional recovery following ICU admission [18]. The investigators randomized 935 patients to either higher dose enteral protein or usual dose enteral protein (the same trial enteral formulae used in TARGET Protein). The primary endpoint was health-related quality of life as measured by the Euro-QoL-5 dimension-5-level questionnaire (EQ-5D-5L) Health Utility Score with between-group differences assessed over three time points using linear mixed-effects models. Trial results have not been published as of July 2024; however, a protocol for a Bayesian re-analysis of the trial data was published in late 2023 [19].

The Replacing Protein Via Enteral Nutrition in Critically Ill Patients (REPLENISH) trial is an open-label, multi-center parallel-group randomized clinical trial to evaluate whether supplemental enteral protein, in addition to standard enteral nutrition to achieve a high enteral protein dose of 2–2.4 g/kg/day, administered from ICU day 5, when compared to usual enteral nutrition to achieve a moderate enteral protein dose of 0.8–1.2 g/kg/day, will reduce all-cause 90-day mortality in critically ill patients [20]. The statistical analysis plan for the REPLENISH trial was recently published [21], and the trial registration site estimates a completion date in mid-2025 (ClinicalTrials.gov ID NCT04475666). The TARGET Protein trial will be a pragmatic trial that will enroll over 3000 patients, the largest protein interventional study conducted in critically ill patients to date, which will provide greater certainty of the effect size protein dosage has on clinical outcomes.

## Methods

TARGET Protein is a cluster randomized, cross-sectional double cross-over open-label trial conducted within eight ICUs across Australia and New Zealand. The study was prospectively registered at the Australian New Zealand Clinical Trials Registry on 1 November 2021 (ACTRN12621001484831).

### Clusters

Four of eight ICUs were randomly allocated to the sequence augmented protein enteral formula alternating after each 3-month period with usual care enteral protein formula; with the other four ICUs following the opposite sequence. Further details regarding treatment allocation generation and randomization are provided in the published study protocol [15]. In the overall 12-month trial duration, this resulted in four periods with alternate enteral nutrition formulae within each of the eight ICU clusters. Patients admitted to each ICU during each 3-month cluster-period received the same trial enteral formula until either the patient reached day 90, was discharged from ICU, or died.

In the cluster double cross-over design, each cluster is randomized to receive each intervention at separate periods of time and individuals are included only once. The randomization of the clusters to the order that the intervention is received enables us to control for period effects. The inclusion of the crossover element increases the power of the study and reduces the required number of participants when compared to a parallel cluster design [22].

### Participants

#### Inclusion criteria

All patients admitted to ICU are eligible if they are:

1.  ≥ 16 years of age
2. Receiving/about to commence EN during their index admission to ICU or receiving/about to commence EN for the first time in ICU during subsequent admissions

#### Exclusion criteria

1. At the commencement of EN, the treating clinician considers the trial enteral formula to be contraindicated.
2. The patient has received greater than 12 h of non-trial enteral formula.
3. The patient has previously participated in the TARGET Protein trial.

**Fig. 1:**
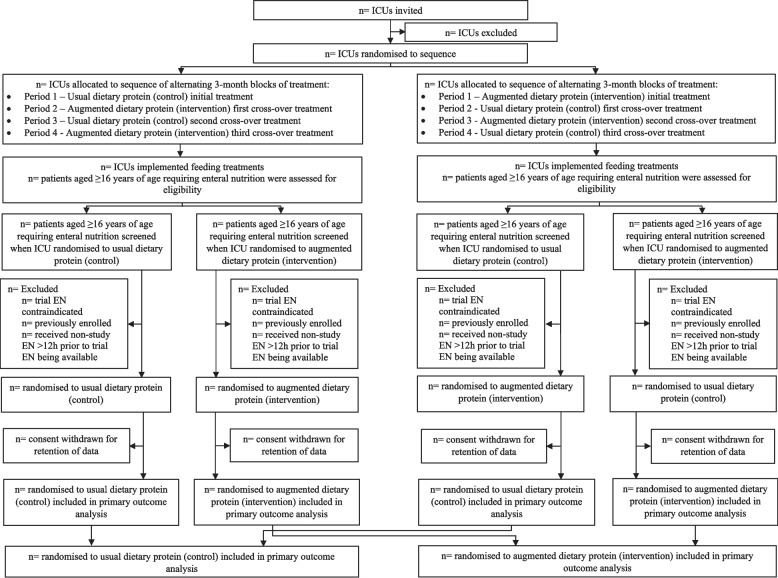
Flowchart showing screening, randomization, and follow-up of participants in the TARGET Protein cluster randomized trial

Patient flow through the trial will be summarized in a Consolidated Standards of Reporting Trials (CONSORT) diagram (Fig. 1). We will report cluster sequence allocation, number of patients assessed for eligibility, number of patients who met trial inclusion and exclusion criteria, number of patients enrolled, number of patients withdrawn, and number of patients included in the primary outcome analysis.

### Intervention and comparator

During the intervention cluster periods (augmented dietary protein), all eligible patients received the enteral formula “Nutrison Protein Intense®” (Nutricia Australia Pty Limited) which contains 100 g protein per 1000 ml and 1260 kcal per 1000 ml. During the comparator cluster periods (usual dietary protein), all patients received the enteral formula “Nutrison Protein Plus®” (Nutricia Australia Pty Limited), which contains 63 g protein per 1000 ml and 1250 kcal per 1000 ml.

### Outcomes

#### Primary outcome

The primary outcome is the number of days free of the index hospital and alive at day 90. This is a patient-centered outcome measure that captures both death and morbidity [23–26].

This will be calculated as 90 days minus all days admitted to the index hospital after commencement of trial enteral formula, minus any days readmitted to the index hospital within 90 days. As patients may be readmitted for part of a day (e.g., for dialysis), a readmission will only be counted when the patient remains in hospital at midnight. Patients will be considered alive if they survive the index-hospital episode, and there is no evidence of death before day 90. In Australia, evidence of death after hospital discharge will be ascertained from the local health record by site investigators and the Australian National Death Index. Similarly, at the New Zealand site, The Australian New Zealand Intensive Care Society Adult Patient Database (ANZICS APD) is linked to the New Zealand national death registry to record death after hospital discharge. Patients who die during the 90-day post-trial recruitment period will be assigned a days free of the index hospital value of 0. Examples of this calculation are provided below:

- A patient who survived and is discharged from hospital on day 30 after commencement of trial enteral formula is assigned a value of 60.
- A patient who survived and is discharged from hospital on day 92 after commencement of trial enteral formula would be censored at day 90 and assigned a value of 0.
- A patient who dies on any day up to and including day 90 is assigned a value of 0.

#### Secondary outcomes

- 1. Days free of the index hospital and alive at day 90 in survivors.

Derived as described for the primary outcome, but the analysis will only be conducted in those patients who survive to day 90 after commencement of trial enteral formula.

- 2. Alive at day 90.

Patients will be considered alive if they are alive at discharge from the index hospital and there is no evidence of death before day 90, either from the local health record or via linkage to the relevant National Death Index. Health service records will be used to provide evidence of patient status, including if the patient was discharged alive from hospital but known to have a health event prior to day 90. Health events include rehabilitation facility admission, outpatient appointment attended, pathology test completed, other medical investigation, or day admission, e.g., dialysis and chemotherapy.

- 3. Duration of invasive ventilation (hours).

Defined as the total hours of invasive mechanical ventilation during the index hospital admission, this includes any re-ventilation periods in ICU following initial weaning during the index hospital admission but does not include ventilation in operating rooms for a procedure. Ventilation during subsequent hospital admissions will not count towards this outcome. Only invasive ventilation will count, and non-invasive ventilation, continuous positive end-expiratory pressure or high-flow oxygen will not contribute to time. The duration of invasive ventilation reported in the Adult Patient Database will be used within this pragmatic registry-based trial noting it will include any periods of time prior to the commencement of study EN. However, the time from ICU admission to commencement of trial enteral nutrition is anticipated to be very short, based on the TARGET trial with similar inclusion criteria [1].

- 4. Duration of admission to ICU (days).

Defined as the time after commencement of trial enteral formula until ICU discharge for the index ICU admission, plus any days that the patient is readmitted prior to hospital discharge, but does not include subsequent hospital admissions.

- 5. Duration of admission to hospital (days).

Defined as the time after commencement of trial enteral formula until hospital discharge for index hospital admission (i.e., it excludes any time spent in hospital if readmitted, which will be counted for the primary outcome).

- 6. Incidence of tracheostomy insertion.

Defined as the proportion of participants requiring new tracheostomy insertion during their study ICU admission.

- 7. Incidence of new renal replacement therapy.

Defined as the proportion of participants commencing new renal replacement therapy during their study ICU admission, subsequent to commencement of trial nutrition.

- 8. Discharge destination.

Discharge destinations are collected in the Adult Patient Database Case Report Form (CRF) as home, other hospital ICU, other acute hospital, rehabilitation, long-term care, and other (a free text field) [15].

### Data source

Data will be sourced from The Australian New Zealand Intensive Care Society Adult Patient Database (ANZICS APD) [27].

In Australia, death after hospital discharge will be ascertained using [1] the local health record where outcome at day 90 is known and [2] via data linkage with the Australian National Death Index. At the New Zealand site, the Adult Patient Database is linked to the New Zealand national death registry to record death after hospital discharge.

Study day 1 commences on the day TARGET Protein trial enteral formula is commenced and concludes at the end of that calendar day (2400 h).

### Sample size

The sample size was calculated using The Shiny Cluster Randomized Trial Calculator and using data from TARGET Calories [1, 28, 29]. In TARGET Calories, the standard deviation for the primary outcome being used in TARGET Protein (days free of the index hospital and alive at day 90) was 11.3 days. Assuming an exchangeable correlation structure, a coefficient of variation for cluster size of 0.5—[based on 8 ICUs (i.e., 8 clusters) each enrolling a mean of 160 patients per 3-month period (95% confidence interval, 80 to 240 patients per cluster per period)]—and the default settings for cluster trials of an intracluster correlation coefficients of 0.02 (with a lower extreme of 0.001 and upper extreme of 0.1), 60 to 65 patients per period per cluster would provide 80% power, and ~ 80 patients per cluster per period would provide 90% power to find a mean difference of 1 day for the primary outcome. Assuming a normal distribution, a sample size of 2560 from the 8 clusters would provide 90% power to detect a mean difference of 1 day free of the index hospital and alive at day 90. However, the primary outcome (days free of the index hospital and alive at day 90) is unlikely to be normally distributed and parametric calculations are optimistic. For this reason, the goal sample size was inflated by 15% to allow for the likely skewed distribution of data, such that ~ 3000 patients from the 8 clusters should provide 90% power [30]. After the trial was completed, it was noted that the sample size estimates specified eight clusters based on eight ICUs being included in the trial. However, the design utilizes four clusters in each of the two treatment sequences. With only four clusters and cluster period size of ≥ 60 patients, there was still > 80% power to detect a 2-day difference in the number of days free of the index hospital and alive at day 90 under a range of plausible assumptions.

### Statistical principles

The statistical analysis will be completed by biostatisticians from the Methods and Implementation Support for Clinical and Health (MISCH) Research Hub (University of Melbourne, Australia). The analyses will be independently checked by a senior biostatistician at MISCH (AK) and any discrepancies between the two analyses will be discussed and resolved by consensus. All analyses will account for the cluster-randomized cross-over design to ensure correct estimation of the 95% confidence intervals (further details provided for the primary and secondary outcomes below). For the primary outcome, estimates, two-sided 95% CIs, and *p* values will be presented. For the secondary outcomes, we will present effect sizes and two-sided 95% CIs only, because the trial was not powered for the secondary outcomes and, thus, have no planned multiplicity adjustment for the secondary outcomes. All statistical tests will be performed in Stata/SE version 18.0 (Stata Corporation, College Station, TX, USA), or R version 4.3.1 or later.

### Analysis sets

Because of the cluster-randomized cross-over design of this trial, the intention-to-treat principle applies at both the cluster level and the participant level. At the cluster level, the intention-to-treat population includes all randomized ICUs that recruited subjects, regardless of the degree of adherence to the study interventions. At the participant level, the intention-to-treat population means that all patients will be analyzed according to the randomized treatment of their ICU at the time of their index ICU admission, regardless of treatment actually received.

### Statistical methods

Descriptive statistics at cluster level will be presented by allocated treatment sequence, and patient characteristics will be summarized by allocated treatment sequence and observation period. Descriptive statistics of variables concerning trial enteral formula delivery (e.g., duration of trial nutrition (hours), mean volume of trial enteral formula delivery (ml/day), calories delivered (kcal/day), and protein delivered (g/day)) will be summarized by allocated treatment sequence. The median (25th and 75th percentiles) of protein (g/day) and calories (kcal/day) from trial nutrition on days 1–5, 10, 20, and 30 of the study will be presented visually by treatment group. The statistical methods for the primary and secondary outcomes are described below. Details regarding the main analysis plan, sensitivity analyses, and subgroup analyses are located in Table 1.

**Table 1:** Analysis plan

Main analysis plan	
**Primary outcome**	
Outcome	Inferential analysis
Number of days free of the index hospital and alive at day 90	Main analysis: Quantile mixed effects model with treatment group, period and delayed start (stratification variable used in randomization) as fixed effects and cluster (ICU) as a random effect
	Secondary analyses: Bayesian quantile mixed model and linear mixed model
**Secondary outcomes**	
Outcome	Inferential analysis
Days free of the index hospital at day 90 in survivors	Quantile mixed effects model
Alive at day 90, incidence of tracheostomy insertion, and incidence of renal replacement therapy	Generalized estimating equations using the ICU as the clustering unit
Duration of invasive ventilation, admission to ICU, and admission to hospital	Time to event (liberation or discharge alive) using Cox regression with covariate adjustment for period and delayed start accounting for mortality as a competing risk
Discharge destination	Descriptive summary only
**Subgroup analyses**	
Planned subgroup analyses will assess heterogeneity of the treatment effect for the primary outcome across the following baseline variables: • Mechanical ventilation • Age 70 years and older • Body mass index 35 kg/m^2^ and greater • New renal replacement therapy—defined as receiving new renal replacement therapy between hospital admission and commencement of trial enteral formula on ICU admission Subgroup analyses will include treatment × subgroup interaction terms in respective regression models	
**Sensitivity analyses**	
The following sensitivity analyses of the primary result will be performed: 1. Exclusion of patients if they are known to have received non-trial enteral formula 2. Exclusion of patients admitted for palliative care or organ donation 3. Imputation of missing data using worst-best and best–worst scenarios (further details provided in section “Handling missing data”)	

*ICU* intensive care unit

#### Primary outcome: number of days free of the index hospital and alive at day 90

##### Main analysis

Comparisons of treatment groups will use individual patient-level data and a quantile mixed effects model will be fitted to compare the median (0.5 quantile) response of the treatment groups. The quantile mixed effects model will include treatment group (augmented protein versus usual care), period (1, 2, 3, and 4), and the stratification variable used in randomization, delayed start (“group 1—commencing May 2022” and “group 2—commencing August 2022”), as fixed effects. Cluster (ICU) will be included as a random effect and assumed to be normally distributed with mean zero and variance component ${\sigma }_{C}^{2}$ [31]. The effect of treatment comparisons will be presented as a difference in medians (95% confidence interval (CI)); the 95% CI will be calculated using the block-bootstrap method [32]. If model convergence issues occur, then the analysis will be performed on the scaled primary outcome (zero mean and unit variance). The treatment effect will then compare the median of the scaled primary outcome between treatment groups.

##### Secondary analyses

A Bayesian quantile mixed model for continuous outcomes will be used to model the median (0.5 quantile) response [33, 34]. The same fixed and random effects described in the main analysis will be included in the Bayesian quantile mixed model. The effect of treatment comparisons will be presented as the posterior mean of the regression coefficient for treatment (difference in medians) and 95% credible interval. Bayesian quantile mixed modeling will be performed using brms package [35, 36] in R. The default hyperprior distributions in the brms packaged will be specified for the fixed effects (improper flat hyperprior) and standard deviation of the cluster random effect (weakly informative half student-t hyperprior).

The following analysis will be performed to align with the sample size calculation and in view of the recent findings from Granholm et al. [37] that linear models adequately estimated group means despite not fitting the data well enough to mimic the “days alive without life support” calculation. Treatment group comparisons will use individual patient-level data and be estimated using a linear mixed effects model. The linear mixed effects model will include treatment group (augmented protein versus usual care), period (1, 2, 3, and 4), and the stratification variable used in randomizsation, delayed start (“group 1—commencing May 2022” and “group 2—commencing August 2022”), as fixed effects. Cluster (ICU) will be included as a random effect and assumed to be normally distributed with mean zero and variance component ${\sigma }_{C}^{2}$. The effect of treatment comparisons will be presented as a mean difference (95% CI) from the linear mixed effects model.

**Secondary outcomes**

##### Days free of the index hospital at day 90 in survivors

This will follow the same approach as for the primary outcome.

##### Alive at day 90, incidence of tracheostomy insertion, and incidence of renal replacement therapy

Comparison of randomized treatment groups will use individual patient-level data and generalized estimating equations (GEE) with a logarithmic link function, binomial distribution, an exchangeable working correlation matrix, and Fay and Graubard’s small sample bias corrected standard errors using the ICU as the clustering unit [31, 38]. Indicator variables for treatment and period will be included in the marginal model, as well as the stratification variable used in randomization, i.e., delayed start. The effect of treatment comparisons will be presented as risk ratio and 95% CI from the GEE analysis [39]. Should the risk ratio model fail to converge, GEE with Poisson distribution, a logarithmic link, exchangeable working correlation matrix, and Fay and Graubard’s small sample bias corrected standard errors will be employed [40].

##### Duration of invasive ventilation, admission to ICU, and admission to hospital

Time to discharge alive from index ICU and index hospital admission, and liberation from invasive mechanical ventilation will be summarized with medians and interquartile ranges obtained from cumulative incidence functions regarding mortality as a competing risk [41].

Treatment group comparisons will use Cox regression with covariate adjustment for period and delayed start, with stratification by ICU, and robust standard errors clustered at ICU level (for any residual within-ICU correlation) to estimate cause-specific hazard ratios and confidence intervals, with patients dying prior to discharge (or extubation) censored at their time of death [41]. Assessment of the proportionality of hazards assumption in these models will be made using Schoenfeld residuals, with resultant covariate stratification or modeling of time-dependent treatment effects, where necessary. For the duration of invasive mechanical ventilation outcome, if the recorded duration of mechanical ventilation for a patient is within 48 h of their duration of hospital stay resulting in death, then it will be assumed that such patients were extubated at that time with palliative intent and, hence, these patients’ data will be censored at time of tracheal extubation in the analyses.

##### Discharge destination

A descriptive analysis will be performed, where the frequency of each discharge destination will be presented by allocated treatment sequence and observation period.

#### Subgroup analyses

Planned subgroup analyses will assess heterogeneity of the treatment effect for the primary outcome across the following baseline variables: mechanical ventilation, age groups (70 years and older vs younger than 70), body mass index (categorized as 35 kg/m^2^ and greater vs less than 35 kg/m^2^), and those with acute kidney injury (AKI; yes vs no), defined as receiving new renal replacement therapy between hospital admission and commencement of trial enteral formula on ICU admission. Subgroup analyses will include treatment × subgroup interaction terms in respective regression models. We will present subgroup specific estimates of the treatment effect (95% CI), which will be derived from the treatment main effect estimate and interaction term estimate and the *p* value from a likelihood ratio test of an interaction between treatment and the subgroup variable.

#### Sensitivity analyses

The following sensitivity analyses of the primary outcome and main analysis will be performed:

1. Exclusion of patients if they are known to have received non-trial enteral formula
2. Exclusion of patients admitted for palliative care or organ donation
3. Imputation of missing data using worst-best and best–worst scenarios (further details provided in section “Handling missing data”)

#### Handling missing data

Given that this trial will be using routinely collected data from the ANZICS APD for the primary and secondary outcomes, we anticipate minimal missing data. However, a sensitivity analysis assessing the impact of missing primary outcome data for the main analysis will be performed and involve imputing outcomes under “worst-best” and “best–worst” case scenarios [42] (see below).

#### Primary outcome

In the “worst-best” scenario, a “worst” outcome event (e.g., 0 days free of the index hospital and alive) will be assigned to all patients missing the outcome in one treatment group, and a “best” outcome event (e.g., 90 days free of the index hospital and alive) will be assigned to all patients missing the outcome in the other treatment group. The “best–worst” scenario is the exact opposite assignment of outcomes.

If substantively different conclusions do not arise from these two analyses, then no further missing data assessments will be performed for that outcome. If a substantively different conclusion does arise, then a more refined analysis will employ a complete case analysis adjusting for baseline covariates predictive of missingness of the specific outcome. These analyses use data from all patients who have complete outcome data and are valid under the “covariate missing at random assumption” that missingness depends on the baseline covariates only and not on the value of the missing outcome itself or of other outcomes [43]. If more than 5% of the data for a primary outcome is missing [42], and when one outcome is missing for a patient but other outcomes are present, further analyses will use multiple imputation methods that take into account the outcomes that are available and the clustered data structure [44].

#### Secondary outcomes

Secondary outcomes will be considered hypothesis generating and will only be analyzed on complete cases. No further analyses or investigation of missing data for secondary outcomes will be conducted.

#### Current status of the trial

The TARGET Protein trial commenced recruitment at four sites (in South Australia, New South Wales, and New Zealand) on 23 May 2022, with four sites in Victoria commencing on 23 August 2022. Each site participated in the trial for 12 months and recruitment ceased on 23 May 2023 and 23 August 2023 respectively. At the time of submission, data linkage was being performed by the Australian Institute of Health and Welfare [45].

## Data Availability

Non-identifiable individual participant data that underlie the results reported in this trial will be made available after 3 years following publication and ending 7 years after publication. Availability will only be made to researchers who provide a written proposal for data evaluation that is judged to be methodologically sound by a committee approved by the TARGET Protein Investigators. If the proposal is approved, access data requestors will be required to sign a data access agreement prior to accessing data.

## Abbreviations

AKI: Acute kidney injury
ANZICS APD: Australian New Zealand Intensive Care Society Adult Patient Database
CONSORT: Consolidated Standards of Reporting Trials
EN: Enteral nutrition
EQ-5D-5L: Euro-QoL-5 dimension-5-level questionnaire
GEE: Generalized estimating equations
ICU: Intensive care units
KDIGO: Kidney Disease: Improving Global Outcomes

## References

[1] Chapman M, Peake SL, Bellomo R, Davies A, Deane A, Horowitz M, et al. Energy-dense versus routine enteral nutrition in the critically ill. N Engl J Med. 2018;379(19):1823–34. 30346225 10.1056/NEJMoa1811687

[2] Puthucheary ZA, Rawal J, McPhail M, Connolly B, Ratnayake G, Chan P, et al. Acute skeletal muscle wasting in critical illness. JAMA. 2013;310(15):1591–600. 24108501 10.1001/jama.2013.278481

[3] Fazzini B, Märkl T, Costas C, Blobner M, Schaller SJ, Prowle J, et al. The rate and assessment of muscle wasting during critical illness: a systematic review and meta-analysis. Crit Care. 2023;27(1):2. 36597123 10.1186/s13054-022-04253-0 PMC9808763

[4] Wieske L, Dettling-Ihnenfeldt DS, Verhamme C, Nollet F, van Schaik IN, Schultz MJ, et al. Impact of ICU-acquired weakness on post-ICU physical functioning: a follow-up study. Crit Care. 2015;19(1):196. 25928709 10.1186/s13054-015-0937-2 PMC4427976

[5] De Jonghe B, Bastuji-Garin S, Durand MC, Malissin I, Rodrigues P, Cerf C, et al. Respiratory weakness is associated with limb weakness and delayed weaning in critical illness. Crit Care Med. 2007;35(9):2007–15. 17855814 10.1097/01.ccm.0000281450.01881.d8

[6] Lee ZY, Ong SP, Ng CC, Yap CSL, Engkasan JP, Barakatun-Nisak MY, et al. Association between ultrasound quadriceps muscle status with premorbid functional status and 60-day mortality in mechanically ventilated critically ill patient: a single-center prospective observational study. Clin Nutr. 2021;40(3):1338–47. 32919818 10.1016/j.clnu.2020.08.022

[7] Compher C, Bingham AL, McCall M, Patel J, Rice TW, Braunschweig C, et al. Guidelines for the provision of nutrition support therapy in the adult critically ill patient: the American Society for Parenteral and Enteral Nutrition. JPEN J Parenter Enteral Nutr. 2022;46(1):12–41. 34784064 10.1002/jpen.2267

[8] Singer P, Blaser AR, Berger MM, Alhazzani W, Calder PC, Casaer MP, et al. ESPEN guideline on clinical nutrition in the intensive care unit. Clin Nutr. 2019;38(1):48–79. 30348463 10.1016/j.clnu.2018.08.037

[9] Murthy TA, Bellomo R, Chapman MJ, Deane AM, Ferrie S, Finnis ME, et al. Protein delivery in mechanically ventilated adults in Australia and New Zealand: current practice. Crit Care Resusc. 2021;23(4):386–93. 38046685 10.51893/2021.4.OA3 PMC10692581

[10] Ridley EJ, Peake SL, Jarvis M, Deane AM, Lange K, Davies AR, et al. Nutrition therapy in Australia and New Zealand intensive care units: an international comparison study. JPEN J Parenter Enteral Nutr. 2018;42(8):1349–57. 29701877 10.1002/jpen.1163

[11] Young PJ, Bellomo R, Chapman MJ, Deane AM, Peake SL. What should we target after TARGET? Crit Care Resusc. 2018;20(4):252–3. 30482131

[12] Bels JLM, Ali Abdelhamid Y, van de Poll MCG. Protein supplementation in critical illness: why, when and how? Curr Opin Clin Nutr Metab Care. 2023;26(2):146–53. 36728596 10.1097/MCO.0000000000000912

[13] Blaauw L, Schoonees A, Robertson N, Visser J. The impact of guideline recommended protein intake on mortality and length of intensive care unit and hospital stay in critically ill adults: a systematic review. Clin Nutr ESPEN. 2024;61:356–68. 38777455 10.1016/j.clnesp.2024.04.003

[14] Lee ZY, Dresen E, Lew CCH, Bels J, Hill A, Hasan MS, et al. The effects of higher versus lower protein delivery in critically ill patients: an updated systematic review and meta-analysis of randomized controlled trials with trial sequential analysis. Crit Care. 2024;28(1):15. 38184658 10.1186/s13054-023-04783-1 PMC10770947

[15] Summers MJ, Chapple LS, Bellomo R, Chapman MJ, Ferrie S, Finnis ME, et al. Study protocol for TARGET protein: the effect of augmented administration of enteral protein to critically ill adults on clinical outcomes: a cluster randomised, cross-sectional, double cross-over, clinical trial. Crit Care Resusc. 2023;25(3):147–54. 37876373 10.1016/j.ccrj.2023.08.001 PMC10581259

[16] Heyland DK, Patel J, Compher C, Rice TW, Bear DE, Lee ZY, et al. The effect of higher protein dosing in critically ill patients with high nutritional risk (EFFORT Protein): an international, multicentre, pragmatic, registry-based randomised trial. Lancet. 2023;401(10376):568–76. 36708732 10.1016/S0140-6736(22)02469-2

[17] Stoppe C, Patel JJ, Zarbock A, Lee ZY, Rice TW, Mafrici B, et al. The impact of higher protein dosing on outcomes in critically ill patients with acute kidney injury: a post hoc analysis of the EFFORT protein trial. Crit Care. 2023;27(1):399. 37853490 10.1186/s13054-023-04663-8 PMC10585921

[18] Bels JLM, Thiessen S, van Gassel RJJ, Beishuizen A, De Bie DA, Fraipont V, et al. Effect of high versus standard protein provision on functional recovery in people with critical illness (PRECISe): an investigator-initiated, double-blinded, multicentre, parallel-group, randomised controlled trial in Belgium and the Netherlands. Lancet. 2024;404(10453):659–69. 39153816 10.1016/S0140-6736(24)01304-7

[19] Heuts S, de Heer P, Gabrio A, Bels JLM, Lee ZY, Stoppe C, et al. The impact of high versus standard enteral protein provision on functional recovery following intensive care admission: protocol for a pre-planned secondary Bayesian analysis of the PRECISe trial. Clin Nutr ESPEN. 2024;59:162–70. 38220371 10.1016/j.clnesp.2023.10.040

[20] Arabi YM, Al-Dorzi HM, Tamim H, Sadat M, Al-Hameed F, AlGhamdi A, et al. Replacing protein via enteral nutrition in a stepwise approach in critically ill patients: a pilot randomized controlled trial (REPLENISH pilot trial). Clin Nutr ESPEN. 2021;44:166–72. 34330462 10.1016/j.clnesp.2021.05.008

[21] Arabi YM, Al-Dorzi HM, Aldibaasi O, Sadat M, Jose J, Muharib D, et al. Statistical analysis plan for the replacing protein via enteral nutrition in a stepwise approach in critically ill patients (REPLENISH) randomized clinical trial. Trials. 2024;25(1):296. 38698442 10.1186/s13063-024-08105-w PMC11064302

[22] Arnup SJ, McKenzie JE, Hemming K, Pilcher D, Forbes AB. Understanding the cluster randomised crossover design: a graphical illustraton of the components of variation and a sample size tutorial. Trials. 2017;18(1):381. 28810895 10.1186/s13063-017-2113-2 PMC5557529

[23] Auriemma CL, Taylor SP, Harhay MO, Courtright KR, Halpern SD. Hospital-free days: a pragmatic and patient-centered outcome for trials among critically and seriously ill patients. Am J Respir Crit Care Med. 2021;204(8):902–9. 34319848 10.1164/rccm.202104-1063PP PMC8534616

[24] Taran S, Coiffard B, Huszti E, Li Q, Chu L, Thomas C, et al. Association of days alive and at home at day 90 after intensive care unit admission with long-term survival and functional status among mechanically ventilated patients. JAMA Netw Open. 2023;6(3):e233265. 36929399 10.1001/jamanetworkopen.2023.3265 PMC10020882

[25] Hodgson CL, Bailey M, Bellomo R, Brickell K, Broadley T, Buhr H, et al. Early active mobilization during mechanical ventilation in the ICU. N Engl J Med. 2022;387(19):1747–58. 36286256 10.1056/NEJMoa2209083

[26] Delaney A, Tian DH, Higgins A, Presneill J, Peake S, Venkatesh B, et al. The association between days alive and out of hospital and health-related quality of life in patients with sepsis. CHEST Critical Care. 2023;1(3).

[27] Australian and New Zealand Intensive Care Society Adult Patient Database (APD) 2024 [Available from: https://www.anzics.org/adult-patient-database-apd/. 10.1016/j.jcrc.2005.11.010 16769456

[28] Deane AM, Little L, Bellomo R, Chapman MJ, Davies AR, Ferrie S, et al. Outcomes six months after delivering 100% or 70% of enteral calorie requirements during critical illness (TARGET). A randomized controlled trial. Am J Respir Crit Care Med. 2020;201(7):814–22. 10.1164/rccm.201909-1810OC 31904995

[29] Hemming K, Kasza J, Hooper R, Forbes A, Taljaard M. A tutorial on sample size calculation for multiple-period cluster randomized parallel, cross-over and stepped-wedge trials using the Shiny CRT Calculator. Int J Epidemiol. 2020;49(3):979–95. 32087011 10.1093/ije/dyz237 PMC7394950

[30] Lehmann EL, D'Abrera HJ. Nonparametrics: statistical methods based on ranks: Holden-day; 2006.

[31] Morgan KE, Forbes AB, Keogh RH, Jairath V, Kahan BC. Choosing appropriate analysis methods for cluster randomised cross-over trials with a binary outcome. Stat Med. 2017;36(2):318–33. 27680896 10.1002/sim.7137

[32] Geraci M, Bottai M. Linear quantile mixed models. Stat Comput. 2014;24(3):461–79.

[33] Congdon P. Quantile regression for overdispersed count data: a hierarchical method. J Stat Distributions Appl. 2017;4(1):18.

[34] Lachos VH, Chen MH, Abanto-Valle CA, Azevedo CL. Quantile regression for censored mixed-effects models with applications to HIV studies. Stat Interface. 2015;8(2):203–15. 26753050 10.4310/SII.2015.v8.n2.a8 PMC4706236

[35] Bürkner P-C. brms: an R package for Bayesian multilevel models using Stan. J Stat Softw. 2017;80(1):1–28.

[36] Bürkner P-C. Advanced Bayesian multilevel modeling with the R package brms. R J. 2018;10(1):395–411.

[37] Granholm A, Kaas-Hansen BS, Lange T, Munch MW, Harhay MO, Zampieri FG, et al. Use of days alive without life support and similar count outcomes in randomised clinical trials - an overview and comparison of methodological choices and analysis methods. BMC Med Res Methodol. 2023;23(1):139. 37316785 10.1186/s12874-023-01963-z PMC10266319

[38] Fay MP, Graubard BI. Small-sample adjustments for Wald-type tests using sandwich estimators. Biometrics. 2001;57(4):1198–206. 11764261 10.1111/j.0006-341x.2001.01198.x

[39] Localio AR, Margolis DJ, Berlin JA. Relative risks and confidence intervals were easily computed indirectly from multivariable logistic regression. J Clin Epidemiol. 2007;60(9):874–82. 17689803 10.1016/j.jclinepi.2006.12.001

[40] Yelland LN, Salter AB, Ryan P. Performance of the modified poisson regression approach for estimating relative risks from clustered prospective data. Am J Epidemiol. 2011;174(8):984–92. 21841157 10.1093/aje/kwr183

[41] Austin PC, Lee DS, Fine JP. Introduction to the analysis of survival data in the presence of competing risks. Circulation. 2016;133(6):601–9. 26858290 10.1161/CIRCULATIONAHA.115.017719 PMC4741409

[42] Jakobsen JC, Gluud C, Wetterslev J, Winkel P. When and how should multiple imputation be used for handling missing data in randomised clinical trials - a practical guide with flowcharts. BMC Med Res Methodol. 2017;17(1):162. 29207961 10.1186/s12874-017-0442-1 PMC5717805

[43] White IR, Carpenter J, Horton NJ. Including all individuals is not enough: lessons for intention-to-treat analysis. Clin Trials. 2012;9(4):396–407. 22752633 10.1177/1740774512450098 PMC3428470

[44] Zhou X, Reiter JP. A note on Bayesian inference after multiple imputation. Am Stat. 2010;64(2):159–63.

[45] Welfare TAIoHa. Data linkage [Available from: https://www.aihw.gov.au/our-services/data-linkage.

